# The potential adverse patient effects of ambulance ramping, a relatively new problem at the interface between prehospital and ED care

**DOI:** 10.4103/0974-2700.43201

**Published:** 2008

**Authors:** Joseph YS Ting

**Affiliations:** Department of Emergency Medicine, Mater Public Hospitals, Raymond Tce, South Brisbane – 4101, Australia. E-mail: joseph.ting@mater.org.au

Sir,

Ambulance ‘ramping,’ when a transported patient has a prolonged wait within the vehicle on arrival at a hospital because there are no vacant care areas or beds available in the hospital's emergency department (ED), is a problem of increasing importance in Australia.[1] Delayed transfer (beyond 8 h) of an admitted patient from the ED to an inpatient unit bed—the so-called access block—contributes to ED overcrowding, to situations where ambulances circle hospitals because they are unable to offload patients and, hence, to adverse patient outcomes.[2]

Another contributor to ambulance ramping and ambulance overcrowding may be the increasing use of ambulances for transport of patients with nonurgent, clinically minor conditions; this can lead to ambulance overcrowding on approach bays to the ED (a phenomenon I will call ‘ED triage access block’). In a single-center study in 2006, the use of ambulances for nonurgent cases significantly increased after patient transport fees were abolished[3]; such measures can be expected to increase already high ambulance transportation rates.[4]

The impact of delayed off-loading and therefore delayed ED care for patients stuck in ramped ambulance queues, as well as the interaction between the urgency of the clinical condition, position in the queue, and patient outcomes, warrants further study. Delay in off-load time has greater clinical consequences for the sicker patients stuck at the end of ambulance queues on arrival to the hospital than for less ill patients located in ambulances ahead of them. Difficulty off-loading patients who need urgent resuscitation results in delayed ED care and worse patient outcomes, especially so in time-critical conditions such as stroke.[5] Moreover, it is not possible to use ambulance crews and vehicles for other jobs whilst they are waiting to off-load a patient.[1]

Patients in an ambulance queue, awaiting off-loading on arrival at the hospital, are not currently subjected to a formalized, well-conducted triage process. Even if ramped ambulance triage were performed, and a sick patient upgraded for more urgent off-loading, repositioning ambulances further ahead in the queue or transferring patients on stretchers from a back-of-queue ambulance to the ED through the confines of a crowded ambulance approach bay poses substantial occupational hazards.

## References

[1] Raabus C Ambulance ramping.

[2] Cameron PA, Campbell DA (2003). Access block: problems and progress. Med J Aust.

[3] Ting JY, Chang AM (2006). Path analysis modeling indicates free transport increases ambulance use for minor indications. Prehosp Emerg Care.

[4] Queensland Treasury Chapter 7: Intersection with the health system. http://www.treasury.qld.gov.au/office/knowledge/docs/qld-ambulance-audit-report/chapter-7-qld-ambulance-audit-report-2007.pdf.

[5] Pepe PE, Zachariah BS, Sayre MR, Floccare D (1998). Ensuring chain of recovery for stroke in your community. Prehosp Emerg Care.

